# Serum-circulating miRNAs predict neuroblastoma progression in mouse model of high-risk metastatic disease

**DOI:** 10.18632/oncotarget.7615

**Published:** 2016-02-23

**Authors:** Satish Kumar Ramraj, Sheeja Aravindan, Dinesh Babu Somasundaram, Terence S. Herman, Mohan Natarajan, Natarajan Aravindan

**Affiliations:** ^1^ Department of Radiation Oncology, University of Oklahoma Health Sciences Center, Oklahoma City, OK, USA; ^2^ Stephenson Cancer Center, Oklahoma City, OK, USA; ^3^ Department of Pathology, University of Texas Health Sciences Center at San Antonio, San Antonio, TX, USA

**Keywords:** serum-circulating miRNA, high-risk metastatic neuroblastoma, miRnome profiling, prognostic biomarker

## Abstract

**Background:**

Circulating miRNAs have momentous clinical relevance as prognostic biomarkers and in the progression of solid tumors. Recognizing novel candidates of neuroblastoma-specific circulating miRNAs would allow us to identify potential prognostic biomarkers that could predict the switch from favorable to high-risk metastatic neuroblastoma (HR-NB).

**Results:**

Utilizing mouse models of favorable and HR-NB and whole miRnome profiling, we identified high serum levels of 34 and low levels of 46 miRNAs in animals with HR-NB. Preferential sequence homology exclusion of mouse miRNAs identified 25 (11 increased; 14 decreased) human-specific prognostic marker candidates, of which, 21 were unique to HR-NB. miRNA QPCR validated miRnome profile. Target analysis defined the candidate miRNAs' signal transduction flow-through and demonstrated their converged roles in tumor progression. miRNA silencing studies verified the function of select miRNAs on the translation of at least 14 target proteins. Expressions of critical targets that correlate tumor progression in tissue of multifarious organs identify the orchestration of HR-NB. Significant (>10 fold) increase in serum levels of miR-381, miR-548h, and miR-580 identify them as potential prognostic markers for neuroblastoma progression.

**Conclusion:**

For the first time, we identified serum-circulating miRNAs that predict the switch from favorable to HR-NB and, further imply that these miRNAs could play a functional role in tumor progression.

## INTRODUCTION

Neuroblastoma (NB), the most life-threatening cancer in infants, accounts for 6% of all cancers in children [1]. About 700 new cases are expected in 2015 [2]. NB is characterized by two discrete disease statuses: 1) favorable disease exhibiting good prognosis with spontaneous regression or differentiation, and 2) high-risk aggressive disease (HR-NB) with systemic metastasis and poor survival [3]. HR-NB is characterized by its extreme heterogeneity, hematogenous metastasis, and 2% long-term survival, despite intensive and multimodal therapy [3]. Understanding the development of HR-NB and identifying novel treatment strategies have become enormous challenges for clinicians and researchers worldwide. Thus, we seek to understand the molecular drivers that orchestrate the switch from favorable NB to HR-NB. The present study aims to find new methods of detecting NB progression at early stages. The current standard diagnostic tools, including blood/urine catecholamine levels, ultrasound, CT, X-Rays, MIBG and PET bone scans, bone marrow aspiration, and biopsy, have been successful in detecting NB. However, these tools are ineffective in diagnosing disease progression from favorable to HR-NB. Hence, there is a critical need for new, minimally invasive diagnostic approaches for the prediction of HR-NB. In the present study, we elaborated the benefits of using serum-circulating micro RNAs (miRNAs) as prognostic markers of HR-NB.

The discovery of small, non-protein coding RNAs, miRNAs, significantly expanded opportunities for the study of tumor biology and cancer diagnostics. miRNAs are typically 22 nucleotides long, non-coding, endogenous, single-stranded RNAs. Thus far, 1,881 unique mature human miRNAs have been identified [4]. Base pairing of the 3′ UTR of the target mRNA with the 5′ end of miRNA causes genomic instability, transcriptional degradation, and translational repression [5]. miRNA can also induce target gene overexpression [6]. miRNAs are frequently located at fragile sites and genomic regions involved in cancer. Their expression is deregulated due to genomic instabilities [7]. While most miRNAs are found within cells, a significant number of miRNAs have been observed outside the cells, including in various body fluids. These miRNAs are stable and display a distinct expression profile [8]. Diagnosis using serum biomarkers is a reliable, less painful technique with demonstrated benefits in multiple tumor systems [9-13]. However, the benefits of such a non-invasive diagnostic tool remain unexplored in NB. In the present study, we investigated the serum-circulating miRNAs that could help diagnose NB progression.

As discussed above, the presence of miRNAs in body fluids may represent an infinite resource of biomarkers in various cancers. Circulating miRNAs have been implicated in the regulation of cancer stem cells [14]. NB is primarily a cancer stem cell tumor [15], presenting with hematogenous metastasis; the highly metastatic aggressive cells that evade multimodal therapy have been shown to possess heightened CSC characteristics [16]. Therefore, recognizing the potential utility of serum-circulating miRNAs in the diagnosis of HR-NB has momentous importance and may improve development of a cure. Serum miRNAs are stable compared with other RNAs, as miRNAs can survive treatment with RNase A for up to three hours or overnight [17]. Repeat freeze-thawing cycles and treatments with low or high pH solutions do not affect serum miRNAs [17]. Circulating miRNAs can remain stable at room temperature for 24 hours and eight freeze-thaw cycles [18]. In addition, circulating miRNAs may selectively modulate the expression of their target oncogenes to prompt metastasis in distant organs [19].

The feasibility of high-throughput approaches allows the precise characterization of miRNAs as prognostic or therapeutic markers for cancer. In the present study, we used a clinically translatable mouse model of human HR-NB and investigated the benefits of serum-circulating miRNAs as predictive diagnostic biomarkers for HR-NB. For the first time, the results of this study identified miRNAs that could be used as biomarkers, and demonstrated their crucial influence on the modulation of the tumor microenvironment at distant sites which could favor HR-NB.

## RESULTS

### Serum-circulating miRNAs can distinguish high-risk metastatic neuroblastoma

**Figure 1:**
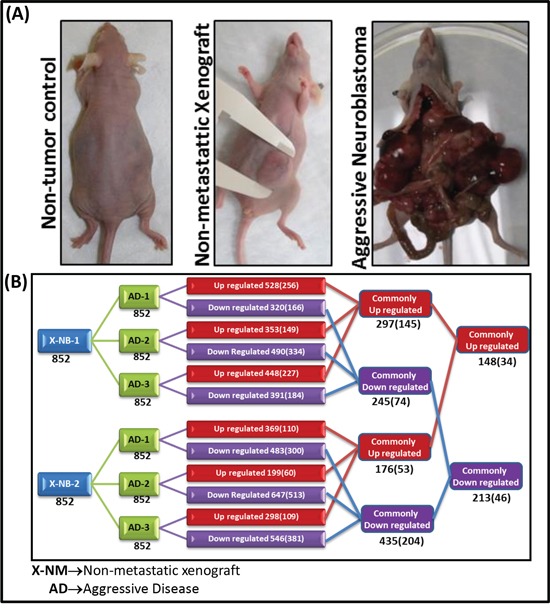
**A.** Photographs of mice representing normal healthy non-tumor controls without treatment, favorable neuroblastoma with primary tumor without metastasis, and high-risk metastatic neuroblastoma with clinically mimicking metastasized tumors in the mediastinum and retroperitoneal, pelvic, abdominal, and chest cavities. **B.** Traverse analysis of whole miRnome expression between animals with favorable non-metastatic primary disease (X-NB) and high-risk metastatic disease (AD). A total of 852 miRNAs were compared between groups. The alterations are color coded (Red – upregulated; Blue – downregulated). The total number of altered molecules under each comparison is provided in the corresponding box. Numbers in parentheses refer to the molecules that are significantly (> or < 2 fold) modulated.

We used whole-miRnome miRNA microarray profiling to assess serum-circulating miRNAs in control mice without tumors, mice bearing non-metastatic human NB xenografts (NM-X), and mice with HR-NB (Figure 1A). The controls were used for baseline mouse background normalization. The non-metastatic neuroblastoma xenografts correlate with favorable human disease. Our mouse model of aggressive metastatic disease develops clinically mimicking organized tumors. Both of these models are well characterized and reported elsewhere [20-22]. In the present study, blank (background) subtracted, spike-in-control normalized expression profiles of serum-circulating miRNAs were compared.

**Figure 2:**
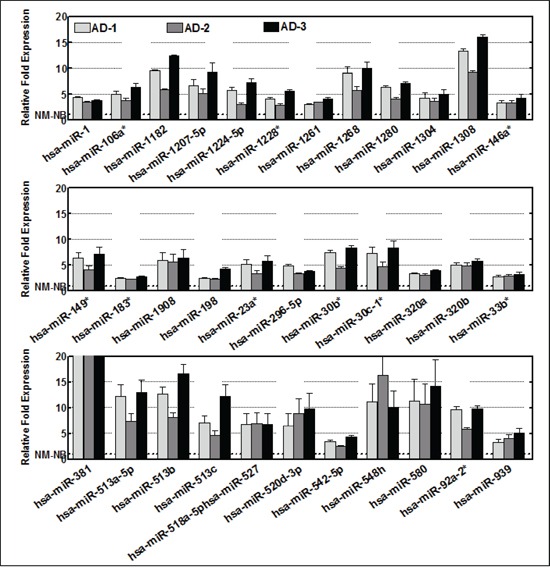
Histograms showing the expression profiles of 34 circulating miRNAs that showed high serum levels (>2 fold) across the animals with high-risk metastatic disease Data mining was performed using traverse analysis comparing the circulating profiles of each non-metastatic primary tumor control to aggressive disease.

First, to eliminate the baseline mouse levels of the serum-circulating miRNA, the expression profiles of non-metastatic and high-risk groups were baseline subtracted to the non-tumor control. The baseline normalized expression was then compared between groups to determine the serum-circulating miRNA(s) that could predict high-risk disease. Traverse analysis between the non-metastatic and HR-NB mice permitted us to eliminate the inter-animal variation in miRNA expression. Overall, we observed an upregulation of 528, 353, and 448 miRNAs in HR-NB mice compared with NM-X1 mice, and an upregulation of 369, 199, and 298 miRNAs when compared with NM-X2 mice (Figure 1B). Interestingly, 297 and 176 of such amplified transcripts remained consistent across HR-NB mice compared with NM-X1 and NM-X2 controls respectively. Distinctly, 148 of these amplified miRNA transcripts were commonly upregulated in HR-NB. Applying stringent criteria (≥2 fold) we observed a significant upregulation of 256, 149, and 227 miRNAs in mice with HR-NB (AD-1, AD-2, AD-3) compared with NM-X1 mice, and 110, 60, and 109 miRNAs in mice with HR-NB (AD-1, AD-2, AD-3) compared with NM-X2 mice. With a total of 145 and 53 miRNAs commonly upregulated in HR-NB, the traverse analysis identified 34 upregulated miRNAs (Figure 2) that could serve as potential prognostic biomarker candidates in this setting.

**Figure 3:**
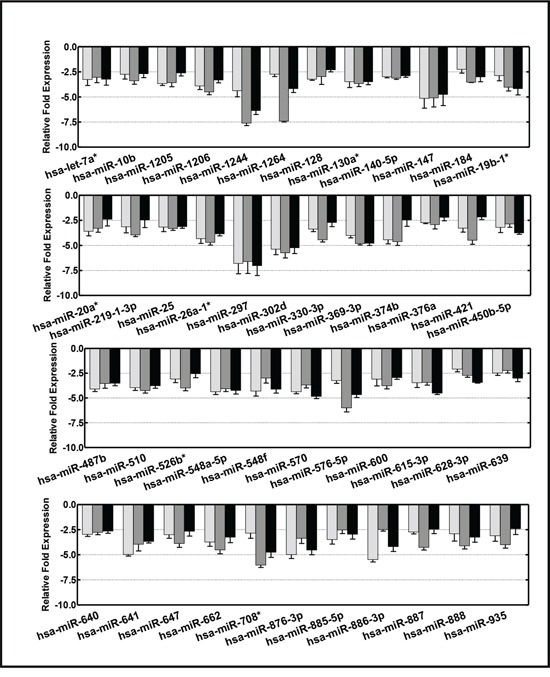
Histograms showing the expression profiles of 46 circulating miRNAs that displayed reduced serum levels (<2 fold) across the animals with high-risk metastatic disease Data mining was performed using traverse analysis comparing the circulating profiles of each non-metastatic primary tumor control to that of aggressive disease.

Since lost or reduced levels of critical serum-circulating molecules may dictate the deregulation of crucial oncogenes, we also examined the miRNAs that were lost in the progression of HR-NB. We observed credible clusters of downregulated miRNA transcripts in NB compared with NM-X1 (320, 490, 391) and NM-X2 (483, 647, 546) mice (Figure 1B). Of these, 166, 334, and 184 miRNA transcripts showed complete (≥2 fold) suppression in HR-NB mice compared with NM-X1 mice. Compared with NM-X2 mice, mice with HR-NB showed 300, 513, and 381 miRNA transcripts with complete (≥2 fold) suppression. A total of 74 and 204 miRNAs remained consistently suppressed across HR-NB mice as opposed to NM-X1 and NM-X2 mice, respectively. More importantly, 46 of these miRNAs were significantly downregulated in all HR-NB mice, but were not downregulated in any controls (Figure 3). The complete loss of select miRNAs is related to the overexpression of their target oncogenes, thus influencing tumor invasion and metastasis.

**Figure 4:**
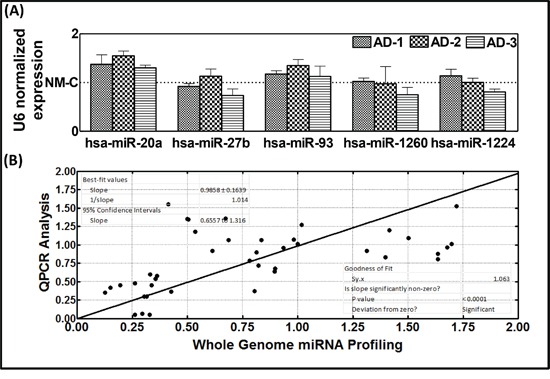
**A.** Histograms of individual miRNA QPCR analysis showing circulating levels of randomly selected miRNAs (miR-20a, miR-27b, miR-1224-3p, miR-1260, and miR-93) in the serum of animals with non-metastatic primary disease or with high-risk metastatic disease. **B.** Correlation analysis of the serum-circulating profiles of miR-20a, miR-27b, miR-1224-3p, miR-1260, and miR-93 observed using the miRnome approach. The individual miRNA-QPCR analysis demonstrates goodness of fit and validates the miRnome expression profiles.

To validate the whole miRnome microarray expression profiles, randomly selected miRNA transcripts in the serum of three different HR-NB mice that were not used for the array studies were analyzed by QPCR. Compared with the non-metastatic favorable disease animals, we observed marginal variations in the expression of miR-20a, miR-27b, miR-93, miR-1260, and miR-1224 (Figure 4A). The QPCR expression profiles of these miRNAs corroborated well with the expression profiles observed using the whole miRnome miRNA array (Figure 4B).

### Defined miRNA candidates as the prognostic biomarkers of HR-NB

Although this unique mouse model of human HR-NB mimics clinical scenarios, and we adopted a mouse base-line normalization approach to eliminate species-associated false positives, the circulation of mouse miRNAs in response to disease progression cannot be ruled out. To better characterize the outcomes observed in the present study and to recognize the defined human miRNAs that can serve as prognostic biomarkers, we performed an extensive mouse-human miRNA homology-based search for significantly upregulated (34) and downregulated (46) miRNAs (Supplementary Table 1). We intentionally avoided all miRNAs that showed any homology between the two species examined, and considered only those miRNAs that had zero homology. This approach allowed us to identify human-specific serum-circulating miRNAs that could serve as prognostic biomarkers for HR-NB. Of the 34 upregulated miRNAs examined, we identified 11 human-specific non-homologous miRNAs, including miR-1261, miR-1268, miR-1280, miR-1304, miR-1308, miR-1908, miR-198, miR-513a-5p, miR-513b, miR-548h, and miR-580 (Supplementary Table 1A). Similarly, of 46 suppressed miRNAs, we recognized 14 human-specific non-homologous miRNAs, including miR-1206, miR-548a-5p, miR-548f, miR-576-5p, miR-600, miR-639, miR-640, miR-641, miR-647, miR-662, miR-886-3p, miR-887, miR-628-3p, and miR-888. As discussed above, suppression of these human-specific miRNAs will later be examined for their effects on the translation of their targets and subsequent disease progression. However, of the 11 identified upregulated non-homologous human specific candidates, miR-548h and miR-580 showed significant increase (≥10 fold) and hence could be used as prognostic biomarkers for HR-NB (Figure 2).

### Critical gene targets of select miRNAs

Connecting serum-circulating miRNAs with molecular responses would allow us to underscore the role of the miRNAs in favoring the niche enrichment at distant sites and cancer metastasis. We examined various gene targets, including predicted and highly predicted targets and experimentally observed targets, for each miRNA in the following four distinct miRNA groups: 1) upregulated non-homologous, 2) upregulated homologous, 3) downregulated non-homologous, and 4) downregulated homologous. Intra-group target data mining identified key gene targets that were commonly regulated by multiple serum-circulating miRNA candidates. The most common gene targets for ‘*upregulated non-homologous miRNAs*’ include TTC39C, ACTR3C, C1orf102, CCPG1, KIAA1328, MAML1, NRXN1, PPP2R3A, PUS10, PWWP2A, SAMD9L, SEC61A2, SF3A1, SLCOA7, ZNF417/ZNF587, ZNF740, and ZSWIM6 (Supplementary Table 2). Similarly, SPTBN1, ARFIP1, FZD3, QKI, MASP1, and VSTM4 comprise the most common gene targets for ‘*downregulated non-homologous miRNAs*’. Conversely, for ‘*upregulated homologous miRNAs*’ BCL2L11, BCL11B, ABL2, BCL11A, ABCA1, ACVR2B, ADAM12, AIF1L, ARHGAP29, ASPH, BET1L, C2orf69, CALM1, CALN1, CCND2, ACAP2, AGO1, ANKRD13A, ANKRD13C, ARHGAP21, ARHGEF18, ASXL2, ATAD3C, ATP5J2-PTCD1, ATP6V1A, BACH2, BDNF, BHLHE22, C7orf43, CELSR3, CNBP, and CCND1 constitute the common gene targets (Supplementary Table 2). Likewise, the common target genes of ‘*downregulated homologous miRNAs*’ included AAK1, ANKRD13C, ACVR1C, ACBD5, AGFG1, AGO1, APPL1, ARHGAP12, CREB1, ADRBK2, AFF3, ANKRD12, ANKRD52, APCDD1, ARID4B, ATG14, ATP11A, BACH2, BAHD1, CREB5, STAT3, BCL11A, CALM1, ABCE1, ACPL2, ADAM12, ADAMTS18, ADAMTS19, AGO4, AGPAT3, APAF1, ARAP2, ARHGAP21, ARHGAP24, ARHGEF12, ARID5B, ARL4A, ARX, ATP13A3, ATP2B2, ATRX, ATXN7L1, BHLHE41, BIRC6, C7orf60, CALB1, KIAA2022, and BCL2L11 (Supplementary Table 2). Further analysis of these targets would provide us with evidence of their functional translation in the progression and distant metastasis of aggressive HR-NB.

### Serum-circulating miRNAs drive the modifications of comprehensive tumor progression signaling pathways

To better underscore the influence of the altered serum-circulating miRNAs in NB with poor prognosis, we examined the interplay of the common gene targets of miRNAs in various cancer progression signaling cascades. A total of 95 of the most common gene targets of the serum-circulating miRNAs were analyzed by *Ingenuity Pathway Analysis* (IPA). IPA analysis revealed that most (74 nos.) of these gene targets were experimentally linked to tumor progression (Supplementary Figure 1). More importantly, these genes were intrinsically involved in 203 canonical signaling pathways that govern tumor progression. The association of these molecules with cancer progression (highlighted in blue) and the overlay of top canonical signaling pathways (linked in gray color) are presented in Supplementary Figure 1. These pathways are significantly linked to cancer cell signaling, cell cycle, cell death and survival, cell morphology, cell-to-cell signaling and interaction, cellular assembly and organization, cellular compromise, cellular development, cellular function and maintenance, cellular growth and proliferation, cellular movement, DNA replication, recombination and repair, free radical scavenging, gene expression, and post translational modification, the hallmarks of sequential events that dictate metastasis and tumor progression (Supplementary Figure 2).

### Alterations in serum-circulating miRNAs corroborate with the modifications of their target proteins' cellular localization and expression in distant sites

**Figure 5:**
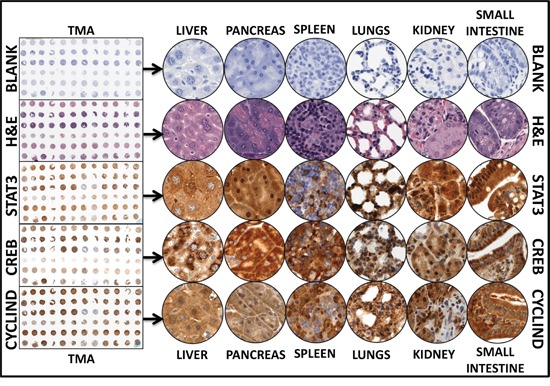
Representative images of the tissue microarray constructed with the replicates of the Liver, pancreas, lungs, kidney, spleens, and small intestine from mice with favorable neuroblastoma and mice with high-risk metastatic disease Automated IHC stained panels (TMA thumbnail and representative field at the magnification of 20X) showing the staining pattern and cellular localization of the serum-circulating miRNAs' protein targets (STAT3, CREB1, CCND1) in Liver, pancreas, lungs, kidney, spleens, and small intestine of mice with favorable neuroblastoma. Representative figures without primary antibody blank controls and H&E stained sections were also included.

**Figure 6:**
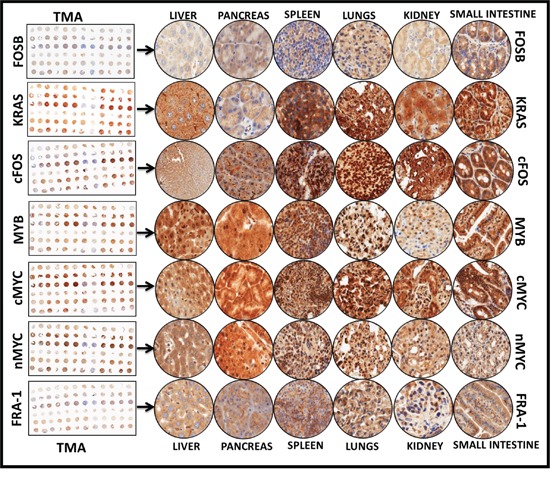
Representative images of the tissue microarray constructed with the replicates of the Liver, pancreas, lungs, kidney, spleens, and small intestine from mice with favorable neuroblastoma and mice with high-risk metastatic disease Automated IHC stained panels (TMA thumbnail and representative field at the magnification of 20X) show the staining pattern and cellular localization of the serum-circulating miRs' protein targets, including MYCN, Myb, FRA-1, FOSB, cMYC, cFOS, and KRAS in Liver, pancreas, lungs, kidney, spleens, and small intestine of mice with favorable neuroblastoma.

To substantiate that the altered levels of serum-circulating miRNAs are related to the modifications of the molecular response that facilitate tumor dissemination, we examined modifications in the expression of randomly selected protein targets that play crucial roles in tumor progression and dissemination. We studied distant organ tissues, including the liver, spleen, pancreas, lungs, kidney, and small intestine, from animals with favorable disease or HR-NB to look for alterations in CREB1, STAT3, Cyclin-D1, MYCN, cMYC, MYB, cFOS, Fosb, kRas and FRA-1 (Figures 5 & 6). To avoid IHC staining inconsistencies across samples, TMA was constructed and the protein localizations were examined with automated IHC coupled with micro digital scanning (Aperio) and spectrum analysis. TMA cores were carefully constructed utilizing histopathological evaluations of individual H&E stained tumor tissues and further confirmed with TMA-H&E staining (Figure 5). Stat3 IHC staining revealed basal levels of positivity in the liver, pancreas, spleen, lung, kidney, and small intestine of NM-X animals (Figure 5). Stat3 positive staining appeared in brown, and was predominantly localized in the nucleus. Compared with the tissues of NM-X animals, we observed a strong Stat3 positivity in the liver and small intestine of animals with HR-NB (Supplementary Figure 3) Conversely, we observed a marginal loss of Stat3 immunoreactivity in the lungs of HR-NB animals. Pancreas, spleens, and kidney from the HR-NB group exhibited variations in intensity of Stat3 expression (Supplementary Figure 3).

Figure 5 presents representative results of IHC for CREB expression in liver, pancreas, spleen, lung, kidney, and small intestine from animals with favorable NB. CREB immunoreactivity was intense in all tissues examined, consistent with the comparable levels reported in normal tissues (www.proteinatlas.org). CREB staining appeared in dark brown and demonstrated ubiquitous nuclear expression. Though we observed a measurable increase in the expression of CREB in all tissues of animals with HR-NB, significant increase is observed only in small intestine (Supplementary Figure 3). Since STAT-3 and CREB are the common targets of downregulated serum-circulating miRNAs, induced expression of STAT3 and CREB validates that the loss of circulating miRNAs dictates target genes' activation in distant organs and may subsequently facilitate metastasis.

Likewise, TMA-IHC analysis of Cyclin-D1 (Ccnd1) revealed a strong immunoreactivity in all tissues of NM-X animals (Figure 5). We observed an intense punctuated cytoplasmic staining (consistent with www.proteinatlas.org) pattern in all six tissues analyzed. In addition, liver, pancreas, spleen, and small intestine also showed moderate to strong nuclear localization. Tissue-specific comparison analysis between favorable and HR-NB mice did not reveal any consistent or significant change in Ccnd1 expression in any tissue (Supplementary Figure 3). Since Ccnd1 is a common target of the upregulated circulating miRNAs, stable regulation without observable activation demonstrates that serum-circulating miRNAs regulate Ccnd1.

MYCN is the most documented critical player in NB genesis and progression. We observed a measurable localization of MYCN in liver, kidney, spleen, pancreas, lung, and small intestine of NM-X animals (Figure 6). Interestingly, we observed a robust and significant MYCN increase in all tissues (except spleen) of HR-NB animals, (Supplementary Figure 4). High levels of MYCN in HR-NB are consistent with published reports and served as positive controls. Liver and kidney of HR-NB animals showed >3 fold increase in MYCN levels (Supplementary Figure 4). Consistently, a significant increase in (*P*<0.001) cMYC expression was observed in HR-NB kidney and liver, with marginal to no modifications in other tissues (Supplementary Figure 4). Notably, kidney from HR-NB animals demonstrated a five-fold amplification of cMYC. Expression patterns of MYB in liver, pancreas, kidney, lung, spleen, and small intestine are presented in Figure 6. Consistent with www.proteinatlas.org, we observed intense nuclear staining and weak punctuated cytoplasmic staining. A robust (*P*<0.001) increase in MYB expression was noted across all tissues in HR-NB animals, compared to tissue-specific expression in NM-X animals (Supplementary Figure 4). IHC revealed a strong cytoplasmic kRAS staining and, the staining was intense in spleens, lungs, and small intestine. Liver and kidney demonstrated moderate expression and pancreas exhibited weak positivity. The tissue-specific expression of kRAS in mice with HR-NB remained comparable to mice with NM-X animals (Supplementary Figure 4).

FOS (cFOS)-IHC revealed intense nuclear staining in spleens, lungs, and kidney from mice with primary disease; pancreas and small intestine showed moderate staining, and Liver displayed weak staining (Figure 6). We observed a strong nuclear positivity of FOSb in lungs, while all other tissues exhibited moderate to weak FOSb expression. We detected moderate expression of FOS-like antigen 1 (FRA-1) in kidney and small intestine, while spleens, Liver, lungs, and pancreas demonstrated weak positivity (Figure 6). Conversely, we observed a significant increase in the expression of cFOS, FOSb, and FRA-1 in the tissues of HR-NB animals (Supplementary Figure 4). Increased cFOS expression remained consistently significant (*P*<0.001) across all tissues examined. In HR-NB animals, Liver, pancreas, and spleens showed robust activation of cFOS. Consistently profound (*P*<0.001) activation of FOSb was evident in Liver, kidney, and small intestine. However, FRA-1 activation was only significant (*P*<0.001) in the kidney of HR-NB animals (Supplementary Figure 4). Together, these data clearly demonstrate the increased expression of key tumor initiation and progression molecules in multiple distant sites of HR-NB animals. Increased expression of these proteins comprising the downstream targets of altered serum-circulating miRNAs allows us to precisely validate the importance of these miRNAs in NB progression.

### Functional verification of the altered serum circulating miRNAs

**Figure 7:**
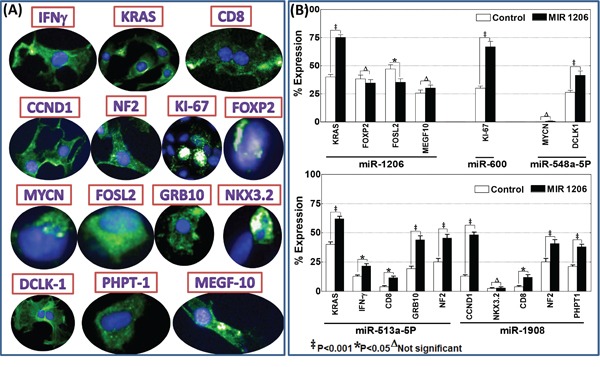
**A.** Operetta high-content confocal immunofluorescence imaging showing localization of IFNγ, kRAS, CD8, CCND1, NF2, KI-67, FOXP2, MYCN, FOSL2, GRB10, NKX3.2, DCLK1, PhPT1 and MEGF10 in parental SH-SY5Y cells. **B.** Histograms of percent expression (mean and standard deviation) of target positivity (A/B*100, where A is the number of Alexa Fluor positive cells and B is the number of total (DAPI) cells per given field) obtained from Columbus analysis showing alterations in the expression of kRAS, FOXP2, FOSL2 and MEGF10 after hsa-miR-1206 inhibition; KI-67 after hsa-miR-600 inhibition; MYCN and DCLK1 (after hsa-miR-548a-5P inhibition; IFNγ, CD8, NF2, GRB10, kRAS after hsa-miR-513a-5P inhibition and, CCND1, NKX3.2, PhPT1, CD8, NF2 after hsa-miR-1908 inhibition. Group-wise comparisons were performed with ANOVA with Tukey's post-hoc correction.

Further, to demonstrate the functional response of the identified serum circulating miRNAs, we utilized miRNA silencing approach and defined the changes in the translation of key protein targets those play in critical roles in various process of metastasis. For this, we selectively silenced five human (non-homologous) specific miRNAs, 2 upregulated (hsa-miR-1908; hsa-miR-513a-5P) and 3 downregulated (hsa-miR-1206; hsa-miR-548a-5P; hsa-miR-600) and examined for the alterations in 14 different critical protein targets including kRAS, CCND1, MYCN, IFNγ, CD8, NF2, KI-67, FOXP2, MYCN, FOSL2, GRB10, NKX3.2, DCLK1, PhPT1 and MEGF10 (Figure 7A). High-content quantitative confocal immunofluorescence analysis revealed that selective inhibition of miR-1206 resulted in significant activation of kRAS (Figure 7B). However, inhibition of miR-1206 did not increase the activation of its other targets tested including FOXP2, FOSL2 and MEGF10 (Figure 7B). Inhibition of miR-600 significantly (*P*<0.001) increased the translation of its target protein KI-67 (Figure 7B). On the other hand, inhibition of miR-548a-5P resulted in significant activation of DCLK1. However, we did not see any activation of MYCN in these cells investigated with and without miR-548a-5P inhibition. Since the cell-line tested is MYCN non-amplified cell and hence the observations serve as the positive controls for the study (Figure 7B). Interestingly, inhibition of miR-513a-5P resulted in the significant activation of kRAS, IFNγ, CD8, GRB10 and NF2 (Figure 7B). Like-wise selective inhibition of miR-1908 demonstrated a significant increase in the expression of its targets CCND1, CD8, NF2 and PhPT1 in this setting. Activated translation of the corresponding protein targets in response to the inhibition of these serum circulating miRNA candidates validates the function of these miRNAs in this setting.

## DISCUSSION

HR-NB remains the leading cause of cancer deaths in infancy. Identifying the molecular drivers that dictate disease progression from favorable NB and the prognostic biomarkers that could predict such progression are the two critical areas that remained underexplored. In this study, we analyzed the serum levels of 852 miRNAs in clinically translatable mouse models of favorable- and HR-NB. Our investigations identified high serum levels of 34 miRNAs and lower serum levels of 46 miRNAs. Though studies have demonstrated the regulatory roles of select miRNAs in NB tumorigenicity, invasion, and metastasis [23-26], this study for the first time illustrates the benefit of using serum-circulating miRNAs as prognostic biomarkers of NB. Such a potential of serum-circulating miRNAs as prognostic biomarkers has been validated beneficial in various other tumor systems [10, 11, 27-30]. More importantly, in this study we identified a cluster of suppressed miRNAs. To that note, the prospective utility of such downregulated miRNAs as prognostic markers has been verified by others in other tumor systems [31]. Moreover, utilizing the whole miRnome approach to examine the serum levels in favorable NB and HR-NB allowed us to quantify up/down-regulated clusters without any equivocation and, identify the clusters that could precisely predict NB progression. Though it is beyond the scope of this manuscript to discuss the importance of each of these miRNA candidates in tumor progression, it is worth mentioning that most of these candidates have been reported in several tumor systems, including NB. For instance, consistent with our observation, increased miR-1 serum levels have been showed in gastric cancer patients [32] and reduced miR-10b levels in colon, renal cancers and melanoma [33, 34].

Since, significant modulation of 80 miRNAs were conserved in HR-NB, despite fail-proof traverse analysis, each of this miRNA may serve as the potential candidate for NB prognostic biomarker. However, one could argue that the observed serum alterations may be associated with stress or tumor burden rather than tumor. To circumvent such a limitation, the data analysis included a mouse-baseline serum level subtraction and further we selected only the candidates that are unique to humans, with zero homology to mouse. Such an approach identified 25 miRNAs (11 up and 14 down) as promising serum prognostic marker candidates that could detect the progression of NB to aggressive metastatic disease. Evidently, except for two candidates in each up (miR-1308, miR-1908) and down (miR-639, miR-628-3p) regulated clusters, none have been reported as serum-circulating diagnostic/prognostic markers in any tumor systems thus far. To that end, studies have shown that miR-1308 may serve as a powerful diagnostic tool for distinguishing healthy controls from patients with pre-cancerous/malignant myeloma [35]. Further, studies have recognized the regulation of serum miR-639 and miR-628-3p in bladder cancer, melanoma, and pancreatic cancer [36-38] and, miR-1908 in nasopharyngeal carcinoma [39].

In addition to their beneficial role as prognostic biomarkers, we examined their potential role in tumor progression. miRNAs function through the regulation of target genes that are mostly downregulated, with miRNA binding during the translational stage. Our target analysis data mining identified many gene drivers of tumor progression. Since it is not feasible to relate the translational response of every target gene, we investigated the key molecular drivers with well documented roles in tumor initiation and progression. First, our results revealed an increased expression of 9 crucial NB tumorigenicity and progression [40-43] related protein targets in multifarious organs of mice with HR-NB. Next, our miRNA silencing studies demonstrated the functional verification for at least 5 miRNAs corresponding to 14 protein targets. More importantly, the results identified three miRNAs, miR-381, miR-548h and miR-580 that exhibited >10-fold increase in mice with HR-NB, which could serve as potential candidates for immediate assessment in HR-NB patients. Though hsa-miR-381 is homologous to mouse miRNA, it has been well studied as a potential marker in other cancer models [44]. For instance, increased expression of miR-381 was associated with cell proliferation and tumor growth; stable expression of miR-381 has been shown to be a critical prognostic biomarker and ideal target in glioma [45]. Circulation of miR-548h and miR-580, the human-specific miRNAs, could be more reliable unique candidates as they are highly upregulated in animals with HR-NB. As discussed above, the expressions of these miRNAs and their utility as prognostic markers remained unexplored.

## MATERIALS AND METHODS

### miRNA isolation and whole miRnome microarray analysis

For this, we utilized the blood samples collected from a well characterized, reproducible mouse model of High-risk aggressive neuroblastoma with metastatic tumors and compared with the non-metastatic primary xenografts [21, 22]. All animal experiments conformed to American Physiological Society Standards for animal care and were carried out in accordance with guidelines laid down by the National Research Council. Protocols were approved by our IACUC. In brief, seven-week-old athymic Ncr-nu/nu nude mice received subcutaneous injections of human NB (SH-SY5Y, 5 × 10^6^) cells suspended in 30% matrigel into their right flanks. Tumor growth was periodically monitored, and tumors were allowed to grow to a volume of approximately 100 mm^3^. These mice served as non-metastatic favorable NB controls. About 30% of the mice that received similar injections developed spontaneous high-risk disease and presented with a manifold of metastatic tumors in various distant sites. Disease progression was vigorous and these mice served as the HR-NB group. Disease origin, progression status, metastatic sites, cytogenetic (whole genome CNV, array CGH)-, transcriptional-, and miRNA-blueprint (whole miRnome), cellular physiognomies, and the reproducibility of the HR-NB model are sequentially characterized and reported elsewhere [20-22].

Blood samples collected from sham, non-metastatic xenograft (NM-X), and HR-NB mice were allowed to clot. The serum was isolated by centrifuging at 2000 rpm at 4°C for 15 min. Isolated clear serum was aliquoted in sterile chilled tubes and stored at −80°C. For miRNA isolation, the serum samples were mixed with 1 ml of Tripure reagent (Roche Diagnostics Corporation, Indianapolis, IN, USA) and chloroform. Samples were then centrifuged and the aqueous phase was collected. After the addition of ethanol to the aqueous phase, total RNA was purified using an RNeasy mini-spin column (Qiagen, Boston, MA, USA). miRNA enrichment was performed using a miRNeasy Mini Kit following the manufacturer's instructions (Qiagen, Boston, MA, USA). The quality and quantity of the isolated miRNA was evaluated using nano drop spectrometry and an Agilent 2100 Bio analyzer (Agilent Technology, Santa Clara, CA, USA). Isolated miRNA (100ng) from each sample were labeled with Cy3 using a Kreatech ULS miRNA labeling kit (Kreatech Diagnostics, Amsterdam, Netherlands). These were hybridized on human miRNA One Array V4 (PhalanxBio, Inc., San Diego, CA, USA) following the manufacturer's protocol. The microarray contains 1,884 unique human miRNA probes and 144 experimental control probes. Each unique probe has three features, and probes contain 100% of Sanger miRBase V18 miRNA content.

After hybridization, the array was washed with 2X SSC wash buffer, scanned using the microarray scanner G2600D (Agilent Technologies, Santa Clara, CA, USA), and quantified using Image Quant (GE Healthcare Bio-Sciences, Pittsburgh, PA, USA). Expression profiles of each feature were background subtracted, normalized to internal U6 feature, and compared between groups using Prism (Graph Pad software, Inc., La Jolla, CA, USA). To exclude any mouse-associated false positives, the experimental groups were baseline corrected with the miRNA expressions observed in sham group. Further, to eliminate inter-animal variations within the group, we adopted NM-X control(s)→HR-NB(s) traverse analysis between every sample analyzed.

### Real-time quantitative miRNA qPCR analysis

The expression levels of miRNA were confirmed with SYBR-Green-based real-time Quantitative PCR (RT-qPCR) using individual miRNA specific primers. For the present study, we used QPCR to confirm expression of selected miRNAs, including hsa-miR-1224-3p, hsa-miR-1260, hsa-miR-27b, hsa-miR-93, and hsa-miR-20a. Hsa-miR-U6 was used as an internal positive control. We reverse transcribed poly (A) tailed (Poly (A) tailing kit, Life Technologies, Grand Island, NY, USA) miRNA using an miRNA EasyScript™ cDNA synthesis kit (Applied Biological Materials, Inc., Richmond, BC, Canada) as per the manufacturer's protocol. QPCR samples were performed in triplicate using a miRNA EasyScript™ cDNA Synthesis Kit (ABM Inc) following the manufacturer's PCR conditions. U6 normalized expression were compared to NM-X controls and expressed as a fold change. Group-wise comparisons were performed with two-way ANOVA with Tukey's post-hoc correction (GraphPad Prism). Similarly, correlations in the expression patterns obtained from the whole miRnome profiling to the QPCR results were examined using GraphPad Prism.

### miRNA target analysis

Data mining applying background subtraction, inter-array normalization, mouse false positive elimination, nullifying inter-animal variation with NM-X vs HR-NB traverse analysis, and stringent selection criteria allowed us to identify clear clusters of upregulated and downregulated serum-circulating miRNAs in HR-NB. These defined miRNAs were further analyzed for their homology with mouse miRNA using a homology search database (http://www.mirbase.org) miRNA target (predicted, most-predicted, and experimentally observed) analysis was performed using an ingenuity pathway analysis (IPA) database. Utilizing IPA (Ingenuity Systems, Inc.), we examined intermolecular interactions and their association with cancer tumor progression. Further, we performed manual crisscross data analysis for the common gene targets across the defined miRNAs to underscore the possible mechanism of distant organ dissemination in this setting.

### Tissue microarray (TMA) construction and automated immunohistochemistry (IHC)

All TMA construction procedures were performed in the Stephenson Cancer Center Cancer Tissue Pathology Core per their standard operating protocols. For the present study, TMA was constructed utilizing the Liver, pancreas, spleens, lungs, kidney, and small intestine of NM-X and HR-NB animals. Duplicate cores were taken from each type of tissue from animals of both groups. These 1.5 mm diameter cores were arrayed, sectioned (4 μm), and baked for subsequent IHC. IHC staining was performed with an automated Leica Bond III according to the manufacturer's protocol using a Bond™ Polymer Refine detection system. For the present study, we used STAT3, Cyclin D1, FRA-1, Fosb, cFOS (all from Santa Cruz Biotechnology, Inc., Dallas, Texas, USA), CREB (Cell signaling technology, Boston, MA, USA), MYCN (Bioss, Inc., Woburn, Massachusetts, USA), Myb, cMYC (Aviva Systems Biology, Corp., San Diego, CA, USA), and KRAS (Proteintech Group, Inc., Chicago, IL, USA) labeling. Appropriate tissue morphologic/pathologic (H&E) control and negative (no primary Ab) controls were examined in parallel. The slides were micro-digitally scanned using an Aperio Scanscope (Aperio Technologies, Inc., Buffalo Grove, IL, USA) slide scanner. We constructed virtual slides with digital histology. This virtual slide technique allows assembly of tissue collections in TMA with variable magnifications. The digital images were then analyzed using Aperio integrated Spectrum (Aperio) software.

### miRNA silencing and quantitative high-throughput confocal immunofluorescence

To verify the function of identified circulating miRNAs on the putative target proteins, we inhibited five human specific miRNAs, 2 upregulated (hsa-miR-1908; hsa-miR-513a-5P) and 3 downregulated (hsa-miR-1206; hsa-miR-548a-5P; hsa-miR-600) and examined for the miRNA-dependent modulations in protein targets. Transient transfection of parental SH-SY5Y cells with hsa-miR-1908-, hsa-miR-513a-5P-, hsa-miR-1206-hsa-miR-548a-5P-, hsa-miR-600-inhibitors (MISSION^®^ Synthetic miRNA Inhibitors, Sigma-Aldrich) were carried out by using Neon electroporation transfection system (Life Technologies). We examined the cellular localization and expression levels of kRAS, FOXP2, FOSL2, MEGF10 (after hsa-miR-1206 inhibition), CCND1, NKX3.2, PhPT1, CD8, NF2 (after hsa-miR-1908 inhibition), IFNγ, CD8, NF2, GRB10, kRAS (after hsa-miR-513a-5P inhibition), KI-67 (after hsa-miR-600 inhibition), MYCN and DCLK1, (after hsa-miR-548a-5P inhibition) in SH-SY5Y cells using Operetta high content quantitative confocal imaging. Paraformaldehyde fixed cells were permeabilized (0.25% Triton X-100), blocked, and labelled with corresponding antibody. They were then tagged with Alexa Fluor 488 conjugated anti-mouse or anti-rabbit secondary antibody (Abcam). The nucleus was counter-labeled with DAPI. At least sixty-three fields/well with a minimum of 3Z planes were analyzed with integrated Columbus image analysis software. Image analysis was performed and the cells-total number, mean/well cell–Alexa Fluor intensity was computed. Group-wise comparisons were performed with ANOVA with Tukey's post-hoc correction (GraphPad Prism).

## CONCLUSION

This study for the first time identified clusters of serum-circulating miRNAs that demonstrated high (34 nos.) and low (44 nos.) serum levels in high-risk aggressive metastatic neuroblastoma. Human-mouse sequence homology identified 25 human specific candidates, of which 21 were unique to the neuroblastoma setting. The results from analysis of the expression of target proteins from distant sites illustrate the functional translation of circulating miRNAs. miRNA inhibition studies verified the function of at least 5 human-specific miRNA candidates. We identified three prominent serum-circulating miRNA candidates which we are currently examining in our laboratory in studies of stage-dependent modulations in the cohorts of patients with high-risk disease.

## SUPPLEMENTARY FIGURES AND TABLES






