# Supplementary material for: Serum-circulating miRNAs predict neuroblastoma progression in mouse model of high-risk metastatic disease
Source: Oncotarget. 2016 Feb 23;7(14):18605–19. doi: 10.18632/oncotarget.7615 (PMC4951313; doi:10.18632/oncotarget.7615)
Supplement: Supplementary file 1 [file oncotarget-07-18605-s001.pdf]

SUPPLEMENTARY FIGURES AND TABLES

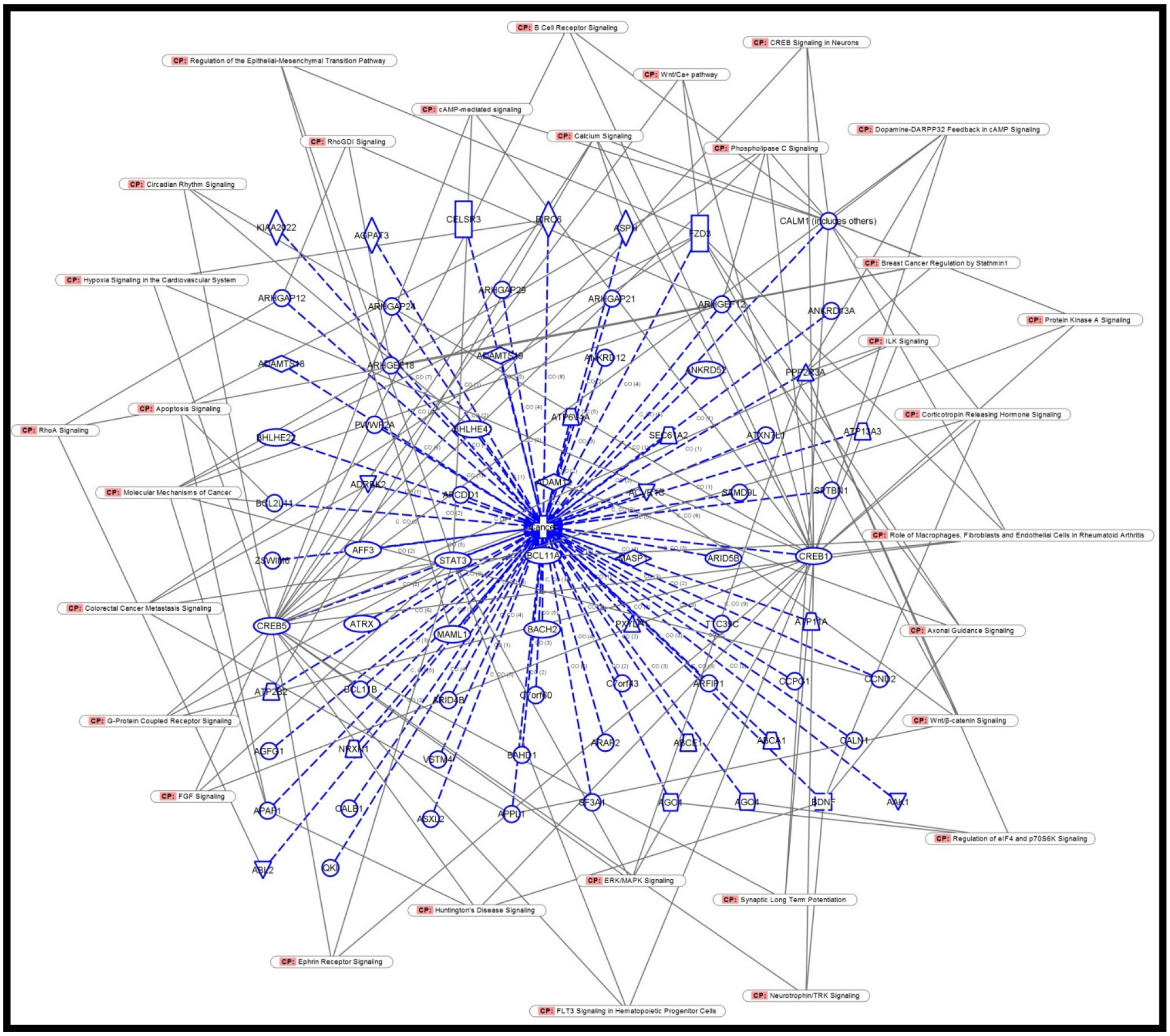

Supplementary Figure S1: Ingenuity pathway analysis of the 95 most common gene targets of the serum circulating miRNAs showing intrinsic associations with cancer. Altogether, 74 genes were experimentally linked to tumor progression. These serum circulating miRNA target genes were critically involved in 203 canonical signaling pathways that govern tumor progression. The association of these molecules with cancer progression (highlighted in blue) and the overlay of top canonical signaling pathways (linked in gray) are presented below.

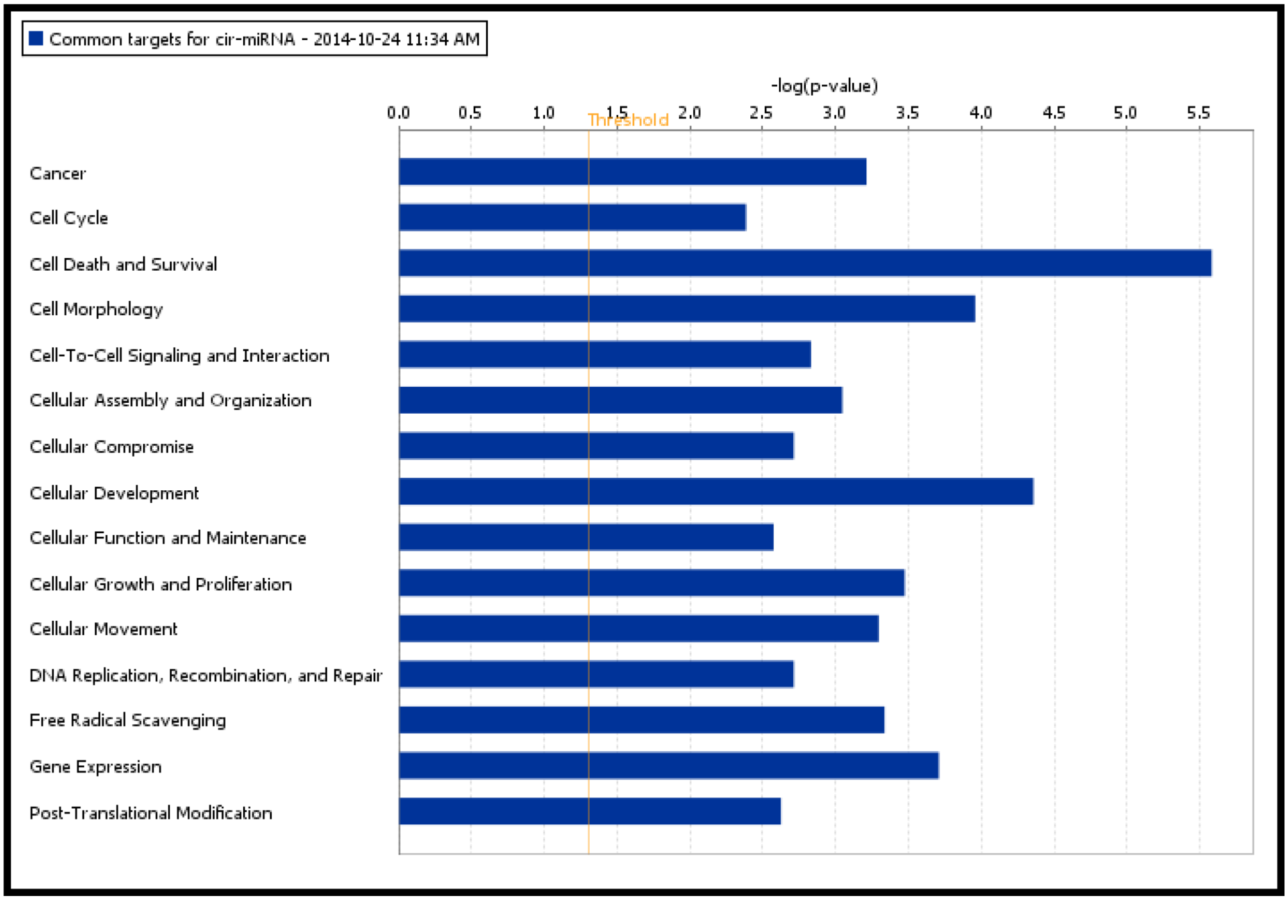

Supplementary Figure S2: Ingenuity pathway analysis of the 95 most common gene targets of the serum circulating miRNAs showing their defined influence in cellular functions, including cancer cell signaling, cell cycle, cell death and survival, cell morphology, cell-to-cell signaling and interaction, cellular assembly and organization, cellular compromise, cellular development, cellular function and maintenance, cellular growth and proliferation, cellular movement, DNA replication, recombination and repair, free radical scavenging, gene expression, and post translational modification, the hallmarks of sequential events that dictate metastasis and tumor progression.

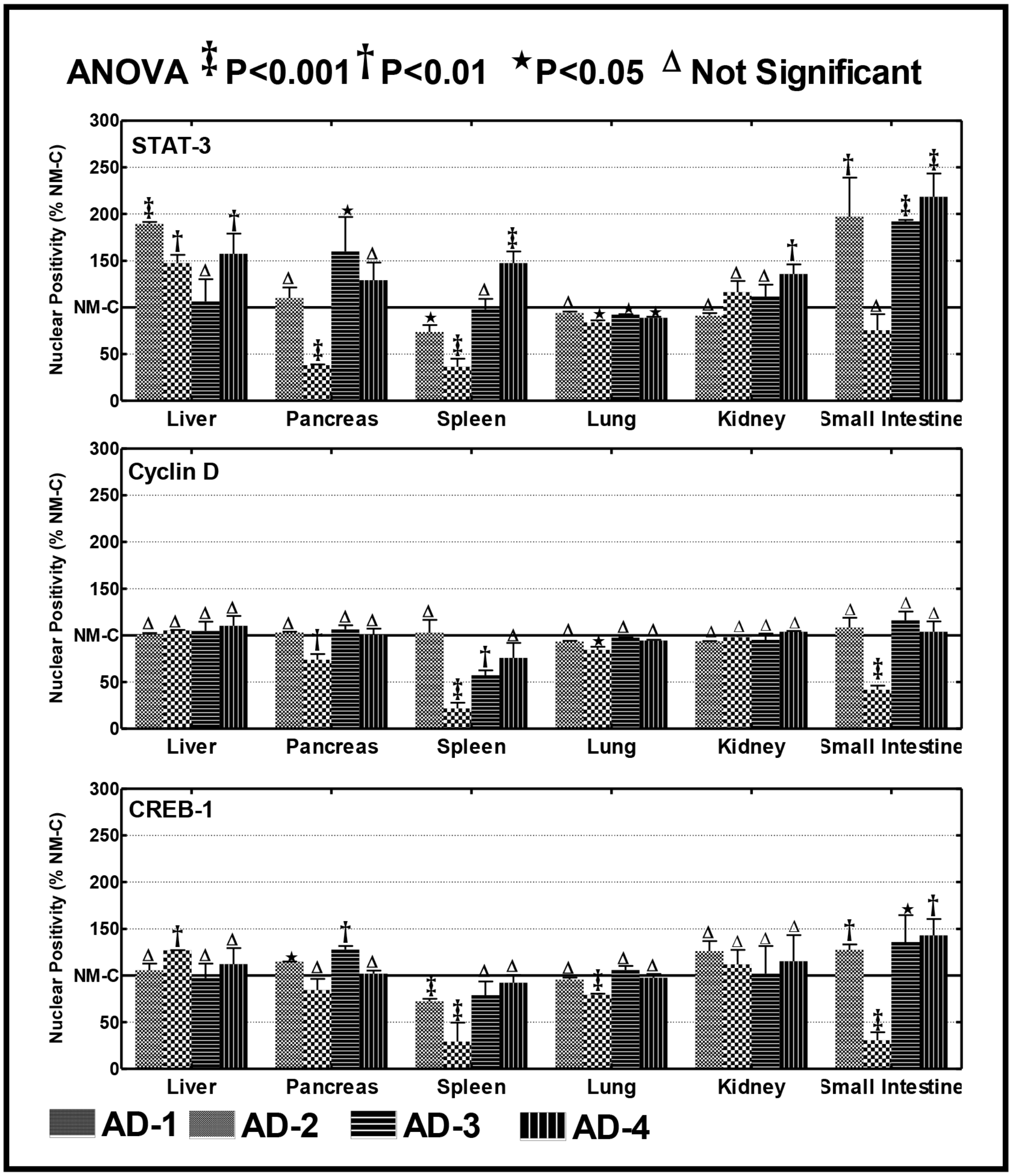

Supplementary Figure S3: Histograms of Aperio-Spectrum immunoreactivity quantification analysis showing expression levels of serum circulating miRNAs' target proteins, including Stat3, Ccnd1, and CREB1 in livers, pancreases, spleens, lungs, kidneys, and small intestines of animals with primary non-metastatic tumor and in animals with high-risk metastatic neuroblastoma. The expression was scored in duplicate cores from four different metastatic tumors and baseline normalized to the average of four cores (from two animals) with favorable disease. Graphed data are the mean expressions with standard deviations. Two-way ANOVA with Tukey's post-hoc correction was performed using GraphPad Prism software.

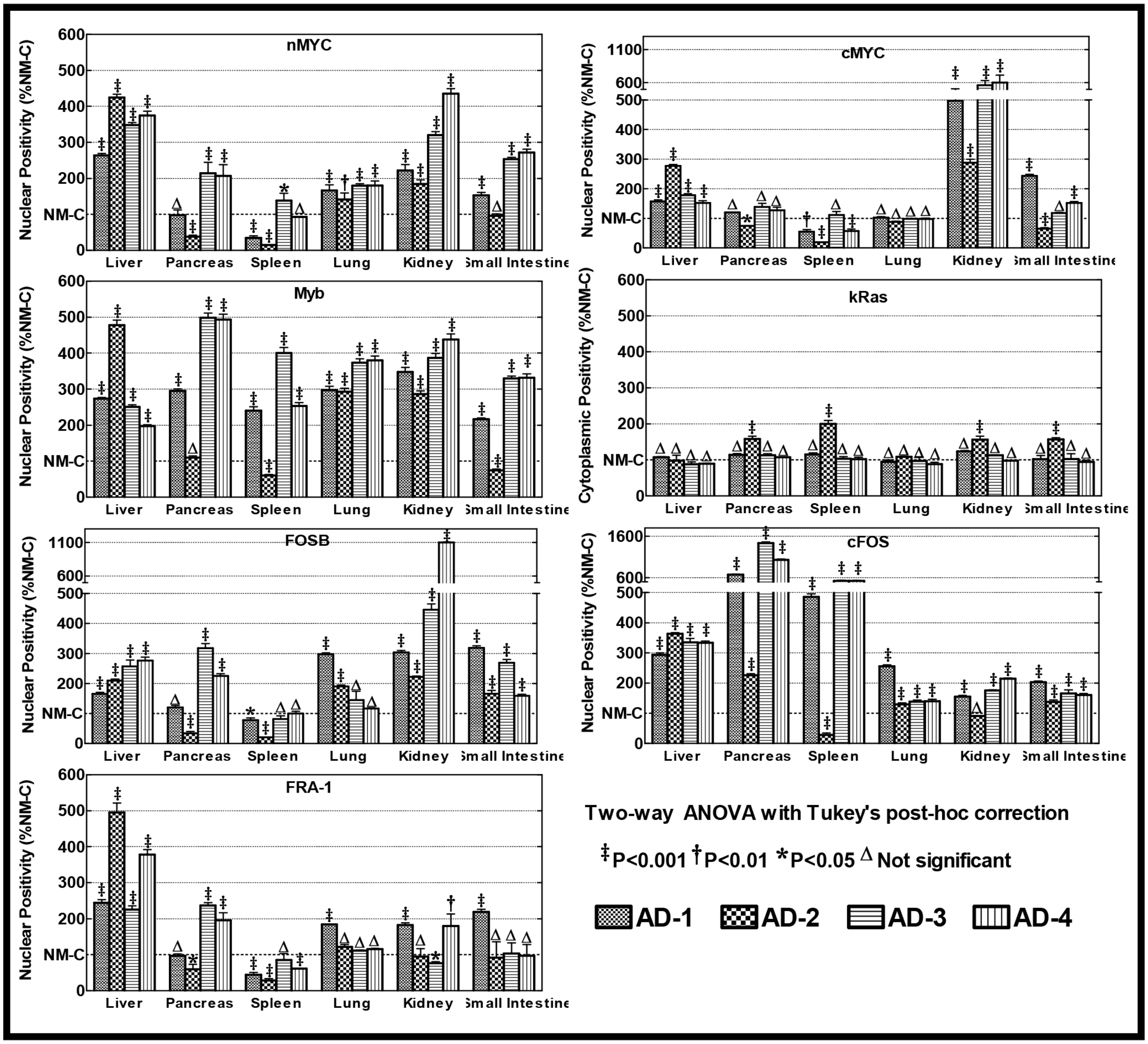

Supplementary Figure S4: Histograms of Aperio-Spectrum immunoreactivity quantification analysis showing expression levels of serum circulating miRNAs' target proteins, including MYCN, cMYC, MYB, kRAS, cFOS, FOSb, and FRA-1 in livers, pancreases, spleens, lungs, kidneys, and small intestines of animals with primary non-metastatic tumor and in animals with high-risk metastatic neuroblastoma. The expression was scored in duplicate cores from four different metastatic tumors and baseline normalized to the average of four cores (from two animals) with favorable disease. Graphed data are the mean expressions with standard deviations. Two-way ANOVA with Tukey's post-hoc correction was performed using GraphPad Prism software.

Supplementary Table S1: Table showing the mouse homologous miRNAs and its alignment scores (http://www.mirbase.org) with the A. significantly upregulated and **B.** suppressed human miRNAs observed in aggressive human neuroblastoma established in athymic nude mice.

See Supplementary File1

Supplementary Table S2: Table showing a list of common targets identified across the serum circulating miRNAs in high-risk metastatic neuroblastoma. Common gene targets for select upregulated and downregulated miRNAs that are homologous and non-homologous are presented in separate clusters.

See Supplementary File 2
