# Supplementary material for: Serum-circulating miRNAs predict neuroblastoma progression in mouse model of high-risk metastatic disease
Source: Oncotarget. 2016 Feb 23;7(14):18605–19. doi: 10.18632/oncotarget.7615 (PMC4951313; doi:10.18632/oncotarget.7615)
Supplement: Supplementary file 2 [file oncotarget-07-18605-s002.docx]

**Supplementary Table 1.** Table showing the mouse homologous miRNAs and its alignment scores ([http://](http://www.mirbase.org/search.shtml)www.mirbase.org) with the **(A)** significantly upregulated and **(B)** suppressed human miRNAs observed in aggressive human neuroblastoma established in athymic nude mice.

**A. Upregulated miRNAs**

| **S. No** | **Human miRNA** | **Homologous mice miRNA** | **Alignment Score** |
| --- | --- | --- | --- |
| 1 | hsa-miR-1 | mmu-miR-1a-3p | 110 |
| 2 | hsa-miR-106a* | mmu-miR-17-5p | 110 |
| 3 | hsa-miR-1182 | mmu-miR-125a-3p | 61 |
| 4 | hsa-miR-1207-5p | mmu-miR-6909-5p | 63 |
| 5 | hsa-miR-1224-5p | mmu-miR-1224-5p | 86 |
| 6 | hsa-miR-1228* | mmu-miR-5130 | 61 |
| 7 | hsa-miR-1261 | Nil | Nil |
| 8 | hsa-miR-1268 | Nil | Nil |
| 9 | hsa-miR-1280 | Nil | Nil |
| 10 | hsa-miR-1304 | Nil | Nil |
| 11 | hsa-miR-1308 | Nil | Nil |
| 12 | hsa-miR-146a* | mmu-miR-146a-5p | 110 |
| 13 | hsa-miR-149* | mmu-miR-149-3p | 96 |
| 14 | hsa-miR-183* | mmu-miR-183-5p | 110 |
| 15 | hsa-miR-1908 | Nil | Nil |
| 16 | hsa-miR-198 | Nil | Nil |
| 17 | hsa-miR-296-5p | mmu-miR-296-5p | 105 |
| 18 | hsa-miR-30b* | mmu-miR-30b-5p | 110 |
| 19 | hsa-miR-30c-1* | mmu-miR-30c-5p | 115 |
| 20 | hsa-miR-320a | mmu-miR-320-3p | 110 |
| 21 | hsa-miR-320b | mmu-miR-320-3p | 101 |
| 22 | hsa-miR-33b* | mmu-miR-33-5p | 82 |
| 23 | hsa-miR-381 | mmu-miR-381-3p | 110 |
| 24 | hsa-miR-513a-5p | Nil | Nil |
| 25 | hsa-miR-513b | Nil | Nil |
| 26 | hsa-miR-513c | mmu-miR-470-3p | 75 |
| 27 | hsa-miR-518a-5p /hsa-miR-527 | mmu-miR-294-3p | 66 |
| 28 | hsa-miR-520d-3p | mmu-miR-294-3p | 83 |
| 29 | hsa-miR-542-5p | mmu-miR-542-5p | 105 |
| 30 | hsa-miR-548h | Nil | Nil |
| 31 | hsa-miR-580 | Nil | Nil |
| 32 | hsa-miR-92a-2* | mmu-miR-92a-2-5p | 87 |
| 33 | hsa-miR-939 | mmu-miR-3057-5p | 68 |
| 34 | hsa-miR-23a* | Mmu-miR-23a-5p | 110 |

| **S. No.** | **Gene Name**  **B. Downregulated miRNAs** | **Homologous mice miRNA** | **Alignment Score** |
| --- | --- | --- | --- |
| 1 | hsa-let-7a* | mmu-let-7a-1-3p | 105 |
| 2 | hsa-miR-10b | mmu-miR-10b-3p | 105 |
| 3 | hsa-miR-1205 | mmu-miR-188-3p | 60 |
| 4 | hsa-miR-1206 | Nil | Nil |
| 5 | hsa-miR-1244 | mmu-miR-6408 | 59 |
| 6 | hsa-miR-1264 | mmu-miR-1264-3p | 101 |
| 7 | hsa-miR-128 | mmu-miR-128-3p | 105 |
| 8 | hsa-miR-130a* | mmu-miR-130a-5p | 80 |
| 9 | hsa-miR-140-5p | mmu-miR-140-5p | 110 |
| 10 | hsa-miR-147 | mmu-miR-147-3p | 91 |
| 11 | hsa-miR-184 | mmu-miR-184-3p | 110 |
| 12 | hsa-miR-20a* | mmu-miR-20a-5p | 115 |
| 13 | hsa-miR-219-1-3p | mmu-miR-219a-1-3p | 101 |
| 14 | hsa-miR-25 | mmu-miR-25-3p | 110 |
| 15 | hsa-miR-26a-1* | mmu-miR-26a-5p | 110 |
| 16 | hsa-miR-297 | mmu-miR-297a-5p | 105 |
| 17 | hsa-miR-302d | mmu-miR-302d-5p | 110 |
| 18 | hsa-miR-330-3p | mmu-miR-330-3p | 106 |
| 19 | hsa-miR-369-3p | mmu-miR-369-3p | 105 |
| 20 | hsa-miR-374b | mmu-miR-374b-5p | 90 |
| 21 | hsa-miR-376a | mmu-miR-376a-3p | 96 |
| 22 | hsa-miR-421 | mmu-miR-421-3p | 115 |
| 23 | hsa-miR-450b-5p | mmu-miR-450b-5p | 101 |
| 24 | hsa-miR-487b | mmu-miR-487b-3p | 110 |
| 25 | hsa-miR-510 | mmu-miR-509-5p | 69 |
| 26 | hsa-miR-526b* | mmu-miR-294-3p | 65 |
| 27 | hsa-miR-548a-5p | Nil | Nil |
| 28 | hsa-miR-548f | Nil | Nil |
| 29 | hsa-miR-570 | mmu-miR-5619-3p | 60 |
| 30 | hsa-miR-576-5p | Nil | Nil |
| 31 | hsa-miR-600 | Nil | Nil |
| 32 | hsa-miR-615-3p | mmu-miR-615-3p | 110 |
| 33 | hsa-miR-639 | Nil | Nil |
| 34 | hsa-miR-640 | Nil | Nil |
| 35 | hsa-miR-641 | Nil | Nil |
| 36 | hsa-miR-647 | Nil | Nil |
| 37 | hsa-miR-662 | Nil | Nil |
| 38 | hsa-miR-876-3p | mmu-miR-876-3p | 101 |
| 39 | hsa-miR-885-5p | mmu-miR-8111 | 61 |
| 40 | hsa-miR-886-3p | Nil | Nil |
| 41 | hsa-miR-887 | Nil | Nil |
| 42 | hsa-miR-888 | Nil | Nil |
| 43 | hsa-miR-19b-1* | mmu-miR-19b-3p | 115 |
| 44 | hsa-miR-628-3p | Nil | Nil |
| 45 | hsa-miR-708 | mmu-miR-708-5p | 115 |
| 46 | hsa-miR-935 | mmu-miR-935 | 115 |
