# Supplementary material for: Serum-circulating miRNAs predict neuroblastoma progression in mouse model of high-risk metastatic disease
Source: Oncotarget. 2016 Feb 23;7(14):18605–19. doi: 10.18632/oncotarget.7615 (PMC4951313; doi:10.18632/oncotarget.7615)
Supplement: Supplementary file 3 [file oncotarget-07-18605-s003.docx]

| **miRNAs** | **Common targets** |
| --- | --- |
| **Upregulated Non-homologous**  hsa-miR-1261, miR-1268, miR-1280, miR-1304, miR-1308, miR-1908, miR-198, miR-513a-5p, miR-513b, miR-548h, miR-580 | TTC39C (4), ACTR3C (3), C17orf102 (3), CCPG1 (3),  KIAA1328 (3), MAML1 (3), NRXN1 (3), PPP2R3A (3), PUS10 (3), PWWP2A (3), SAMD9L (3), SEC61A2 (3), SF3A1 (3), SLCOA7 (3), ZNF417/ZNF587 (3), ZNF740 (3), ZSWIM6 (3) |
| **Downregulated Non-homologous**  hsa-miR-1206, miR-548a-5p, miR-548f, miR-576-5p, miR-600, miR-639, miR-640, miR-641, miR-647, miR-662, miR-887, miR-886-3p, miR-888, miR-628-3p | SPTBN1 (5), ARFIP1 (4), FZD3 (4), QKI (4), MASP1 (4),  VSTM4 (4), 67 targets (3) |
| **Upregulated homologous**  hsa-miR-1, miR-106a*, miR-1182, miR-1207-5p, miR-1224-5p, miR-1228*, miR-146a*, miR-149*, miR-183*, miR-296-5p, miR-30b*, miR-30c-1*, miR-320a, miR-320b, miR-33b*, miR-381, miR-513c, miR-518a-5p, miR-520d-3p, miR-542-5p, miR-92a-2*, miR-939, miR-23a* | BCL2L11 (8), BCL11B (7), ABL2 (6), BCL11A (6), ABCA1 (5), ACVR2B (5), ADAM12 (5), AIF1L (5), ARHGAP29 (5), ASPH (5), BET1L (5), C2orf69 (5), CALM1 (5), CALN1 (5), CCND2 (5), ACAP2 (4), AGO1 (4), ANKRD13A (4), ANKRD13C (4), ARHGAP21 (4), ARHGEF18 (4), ASXL2 (4), ATAD3C (4), ATP5J2-PTCD1 (4), ATP6V1A (4), BACH2 (4), BDNF (4), BHLHE22 (4), C7orf43 (4), CELSR3 (4), CNBP (4), CCND1 (4) |
| **Downregulated homologous**  hsa-let-7a*, miR-10b, miR-1205, miR-1244, miR-1264, miR-128, miR-130a*, miR-140-5p, miR-147, miR-184, miR-20a*, miR-219-1-3p, miR-25, miR-26a-1*, miR-297, miR-302d, miR-330-3p, miR-369-3p, miR-374b, miR-376a, miR-421, miR-450b-5p, miR-487b, miR-510, miR-526b*, miR-570, miR-615-3p, miR-876-3p, miR-885-5p, miR-19b-1*, miR-708, miR-935 | AAK1 (8), ANKRD13C (8), ACVR1C (7), ACBD5 (6), AGFG1 (6), AGO1 (6), APPL1 (6), ARHGAP12 (6), CREB1 (6), ADRBK2 (5), AFF3 (5), ANKRD12 (5), ANKRD52 (5), APCDD1 (5), ARID4B (5), ATG14 (5), ATP11A (5), BACH2 (5), BAHD1 (5), CREB5 (5), STAT3 (5), BCL11A (5), CALM1 (5), ABCE1 (4), ACPL2 (4), ADAM12 (4), ADAMTS18 (4), ADAMTS19 (4), AGO4 (4), AGPAT3 (4), APAF1 (4), ARAP2 (4), ARHGAP21 (4), ARHGAP24 (4), ARHGEF12 (4), ARID5B (4), ARL4A (4), ARX (4), ATP13A3 (4), ATP2B2 (4), ATRX (4), ATXN7L1 (4), BHLHE41 (4), BIRC6 (4), C7orf60 (4), CALB1 (4), KIAA2022 (4), BCL2L11 (4) |

**Supplementary Table 2:** Table showing a list of common targets identified across the serum circulating miRNAs in high-risk metastatic neuroblastoma. Common gene targets for select upregulated and downregulated miRNAs that are homologous and non-homologous are presented in separate clusters.
